# Supplementary material for: Reinforcing a Thermoplastic Starch/Poly(butylene adipate-co-terephthalate) Composite Foam with Polyethylene Glycol under Supercritical Carbon Dioxide
Source: Polymers (Basel). 2022 Dec 28;15(1):129. doi: 10.3390/polym15010129 (PMC9824321; doi:10.3390/polym15010129)
Supplement: Supplementary file 1 [file polymers-15-00129-s001.zip › polymers-2040902-Supplementary.pdf]

Supporting Information

Reinforcing a Thermoplastic Starch/Poly(butylene adipate-co-terephthalate) composite foam with Polyethylene Glycol under Supercritical Carbon Dioxide

Chih-Jen Chang [1,†], Jayashree Chandrasekar [1,†], Chia-Jung Cho [2,*,†], Manikandan Venkatesan [1], Pin-Shu Huang [2], Ching-Wei Yang [1], Hsin-Ta Wang [1], Chang-Ming Wong [3] and Chi-Ching Kuo [1,*]

[1] Institute of Organic and Polymeric Materials, Research and Development Center of Smart Textile Technology, National Taipei University of Technology, Taipei 10608, Taiwan
[2] Institute of Biotechnology and Chemical Engineering, I-Shou University, Kaohsiung 84001, Taiwan
[3] CoreTech System Co., Ltd., Hsinchu 30265, Taiwan
* Correspondence: ppaul288@isu.edu.tw (C.-J.C.); kuocc@mail.ntut.edu.tw (C.-C.K.)
† These authors contributed equally to this work.

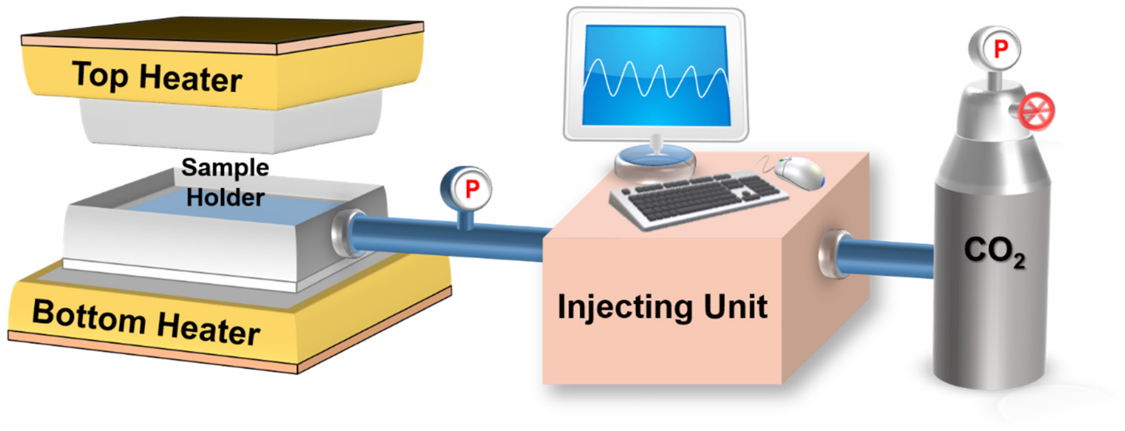

Figure S1. Schematic representation of foaming system, the processing system is used for CO_2 batch foaming.

Supercritical Batch Foaming Process

Figure S1 schematically describes we used the processing system, for CO_2 batch foaming in a previous study. An upper and under part of a batch die is mounted on the upper and under the platform of the press, respectively. A small tunnel in the batch die was connected with a CO_2 injection unit by a stainless steel tube. The press can provide heat to raise the temperature of the batch die and force it to seal foaming agent CO_2 in the die. The inside dimensions for the batch die are 250 mm in diameter and 13 mm in height. The foaming procedure was as follows: (a.) PBAT with thermoplastic starch and silane/PEG were blended to form a large sheet with 3 mm in thickness. Next, the large sheet was cut into several small square samples with 25 mm in length and as well as 3

mm in width thickness for foaming. (b.)The batch die was heated to reach setting temperatures ranging from 80 °C to 105 °C first; then five square samples were put in the under part of the die, and next seal the upper and under part of dying by the force from the piston of the press. (c.) Foaming agent CO_2 was injected into the batch die by using CO_2 injection unit through stainless steel tube until the setting pressure which is 17 MPa or 23.8 MPa. (d.) The five square samples were soaked with CO_2 under a supercritical condition in an hour to let samples reach saturation condition. (e.) The batch die was opened after an hour and the open rate of the die is instant less than 1 second by lowering the piston of the press. (f.) The five foam samples were taken out immediately from dying to prevent shrinkage from a high temperature. (g.) The foam samples were cooled at room temperature to let foam samples be stable. (h.) The properties and structures of five foam samples at the same foaming conditions were evaluated when foam samples are at room temperature after 24 hours.

The preparation process of TPS detailed information

(A). Functional group chemically modified TPS experiment

To carry out the experiment of chemically modifying TPS with functional groups, first, commercially available starch raw material, water, and appropriate glycerin are kneaded in the kneader (Knead Machine), where the starch is uniformly thermally

plasticized into TPS. Then a functional group modifying agent (coupling agent) is added to modify TPS. The functional group modifying agent includes Silane and PEG with four different molecular weights, The reaction temperature is 70-55 °C and the reaction time is 30-60 min. The granulator performs modification and granulation to produce modified TPS particles.

(B). Mixing experiment of modified TPS/biodegradable polyester composite

The modified TPS is added with different proportions of biodegradable polyester PBAT and mixed with the plastic spectrometer mixing machine so that TPS and biodegradable polyester are uniformly mixed and dispersed to form a compound. The temperature of the plastic spectrometer mixing chain equipment was set at 90~145 °C, screw speed at 50~100 rpm for re-granulation.

(C). Production of modified TPS/biodegradable polyester foam test piece

The modified TPS/biodegradable polyester composite particles enter the hot press machine, the temperature of the equipment is set at 140~165 °C, and the square test pieces with the size of 75 mm × 75 mm × 3 mm are hot pressed.

(D). Supercritical foaming experiment of modified TPS/biodegradable polyester composite

Put the hot-pressed 75 mm × 75 mm × 3 mm laminated square test piece into the mold of the supercritical foaming equipment, and obtain different foams according to different foaming parameter settings. The production parameters are as follows: The foaming temperature is set at 80~105 °C, the foaming pressure is 17 MPa and 23.8 MPa, and the CO_2 impregnation time is 60 minutes. This impregnation time can cause the CO_2 in the material to reach a saturated state.

(E). Analysis of specific gravity, appearance, and internal structure of modified TPS/biodegradable polyester foam

Observation of SEM foam structure type -The foam sample after supercritical foaming is plated with gold on the cross-section by vacuum evaporation, and then the structure of the foam cells and the size of the bubbles are observed and analyzed by SEM. Dispersion in the composite.

(F). Modified TPS/biodegradable polyester composite for mechanical property test

The modified TPS/biodegradable polyester composite particles enter the hot press machine, the temperature of the equipment is set at 140~165 °C, and the test piece is

hot pressed to a size of 75mm × 75mm × 3mm. The tensile speed of the universal tensile machine is 50 cm/min, and the load is 5 KG for the mechanical property test.

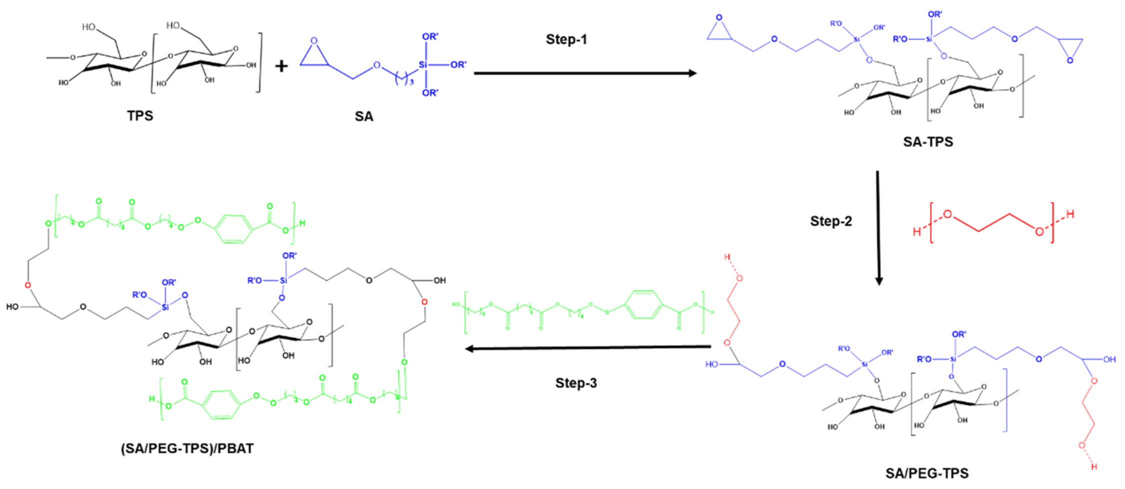

Figure S2. Chemical structure of foam forming process.

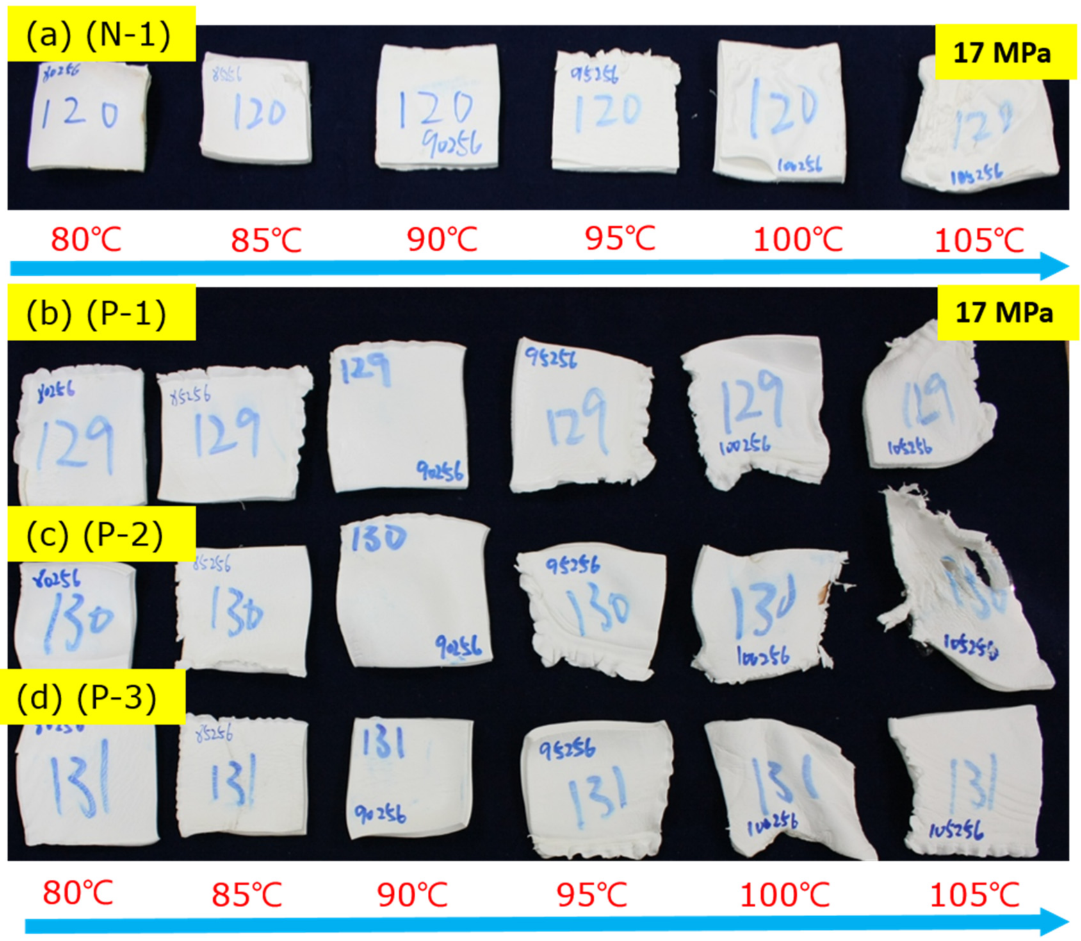

Figure S3. Dependence of TPS/PBAT and (PEG-TPS)/PBAT foam appearance on six

foaming temperatures and the foaming pressure of 17 MPa. (a) [N-1], (b) [P-1], (c) [P-2], and (d) [P-3].

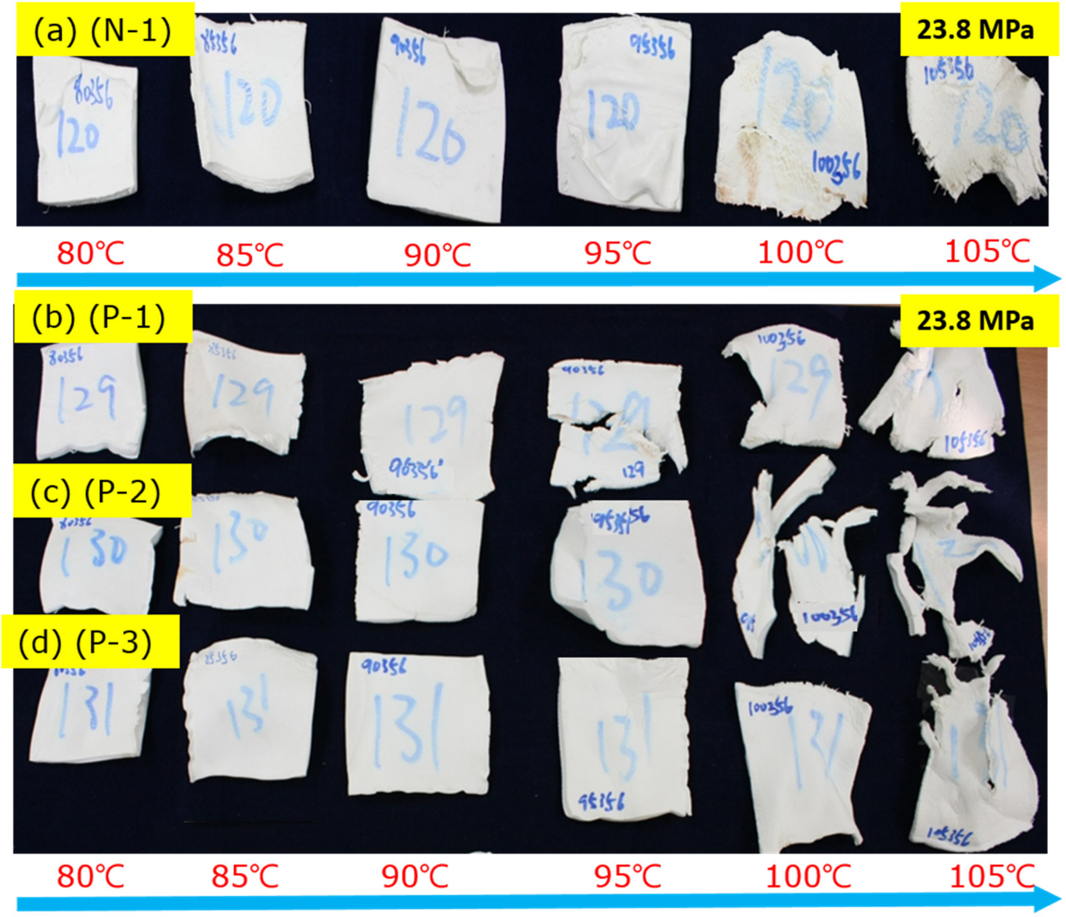

Figure S4. Dependence of TPS/PBAT and (PEG-TPS)/PBAT foam appearance (a) [N-1], (b) [P-1], (c) [P-2], and (d) [P-3] on six foaming temperatures and the foaming pressure of 23.8 MPa.

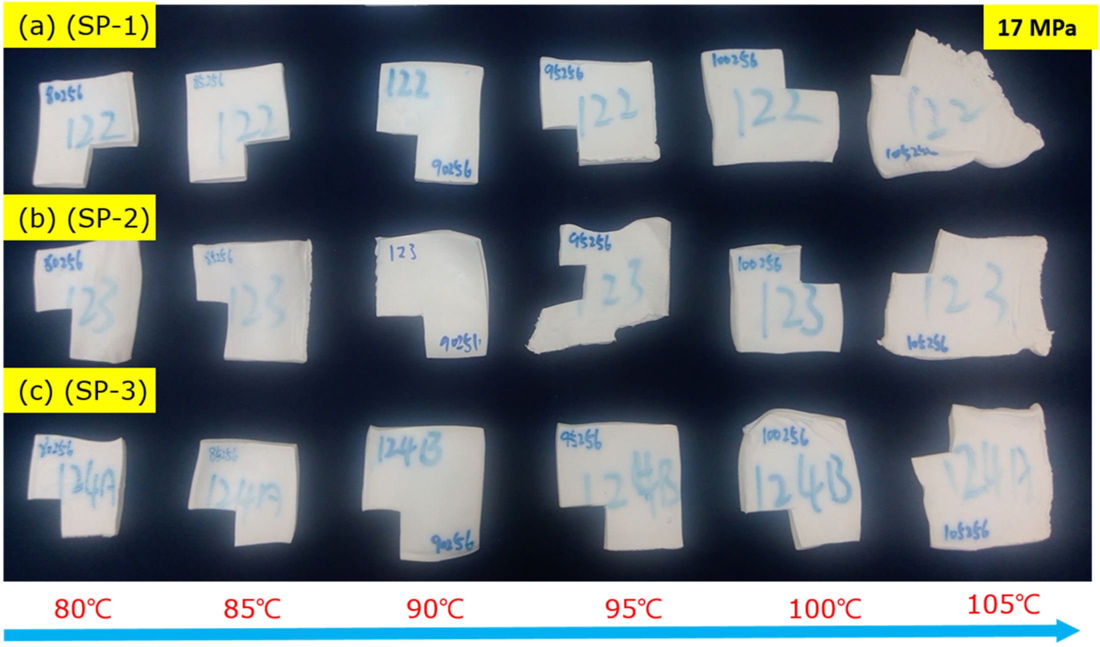

Figure S5. Dependence of (PEG-TPS with SA)/ PBAT foam appearance on six foaming temperatures and the foaming pressure of 17 MPa. (a) [SP-1], (b) [SP-2], and (c) [SP-3].

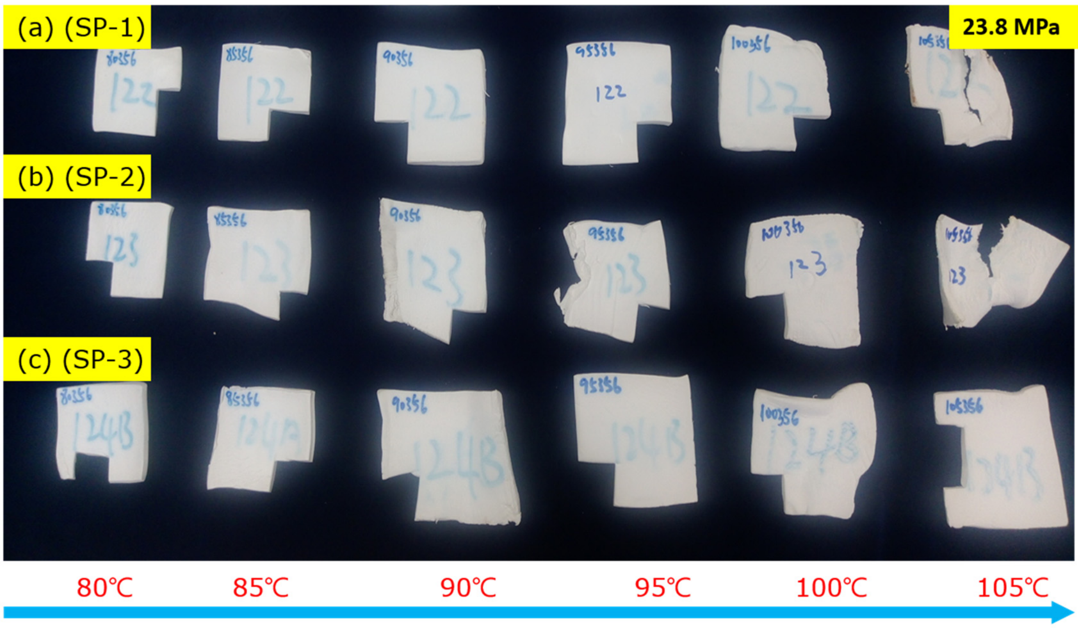

Figure S6. Dependence of (PEG-TPS with SA)/ PBAT foam appearance (a) [SP-1], (b) [SP-2], and (c) [SP-3] on six foaming temperatures and the foaming pressure of 23.8 MPa.

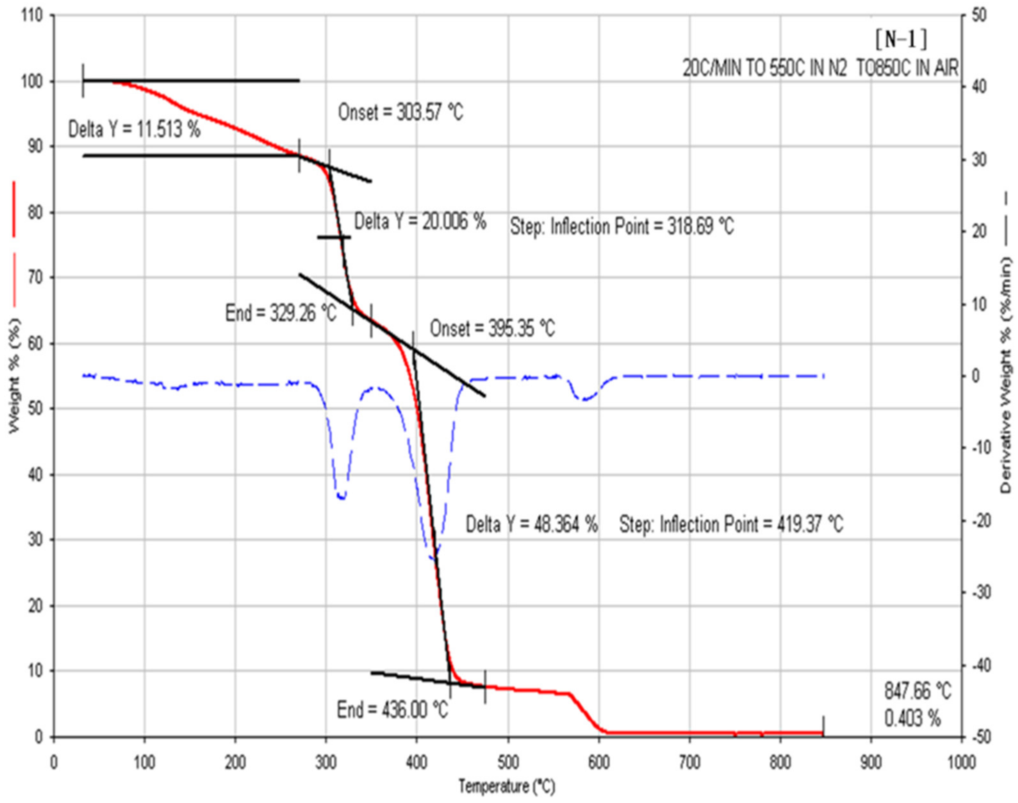

Figure S7. TGA of [N-1]. The first thermal degradation occurs at the temperature of 303.57°C, the mass loss is about 11% in the pyrolysis temperature range of low-molecular glycerol, and it is biodegradable at 350°C~436°C with mass loss of polymer PBAT about 48%.

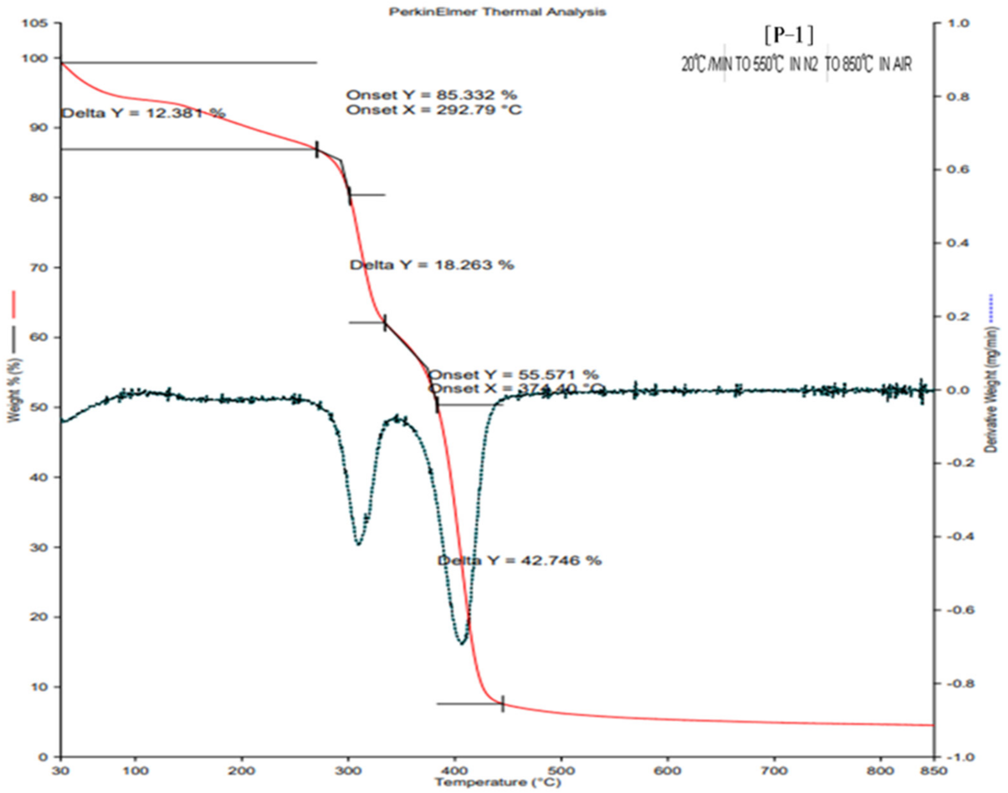

Figure S8. TGA of [P-1]. The first thermal degradation occurs at 292.79 ˚C. Before that, the mass loss of glycerol and PEG is about 12.3%. 292–374 ˚C is the thermal degradation temperature of TPS, 350~436 ℃ is the pyrolysis temperature range of biodegradable polymer PBAT, and the heat loss is about 42.7%.

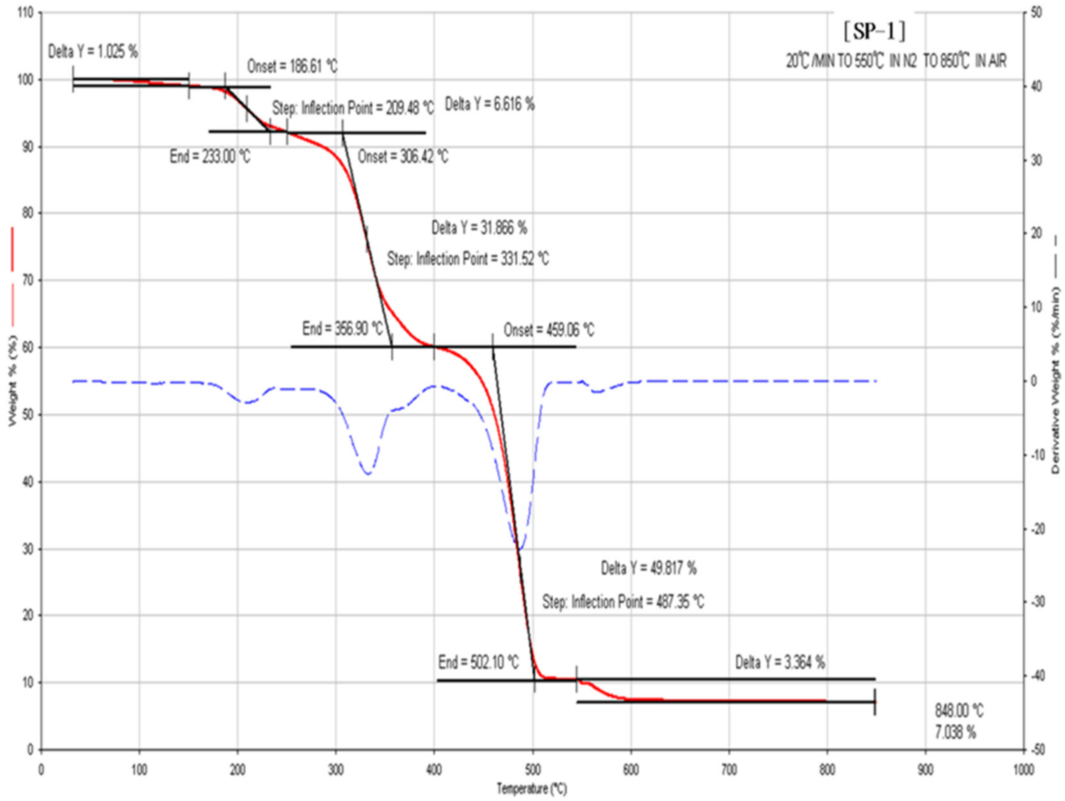

Figure S9. TGA of [SP-1]. The thermal degradation began to occur at 186 °C. The thermal degradation continued upto the temperature of 209 °C with weight loss of 6.6%. The second degradation occurred at the temperature of 306.42 ˚C. It can be seen that the heat resistance of TPS[P-1] after adding SA is 14 °C higher than that of PEG1000[P-1], and it is found that the initial degradation temperature of the biodegradable polymer PBAT has increased from 350 °C to 400 °C, and the final thermal degradation has been increased from 450 °C to 500 °C. It can be seen that the addition of SA not only increase the heat resistance temperature of TPS, but also increase the heat resistance temperature of PBAT. The reason is that SA is an epoxy-based modifier and its structural thermal stability is better. It can also increase the compatibility of TPS and PBAT and generate more entanglement characteristics between molecules.

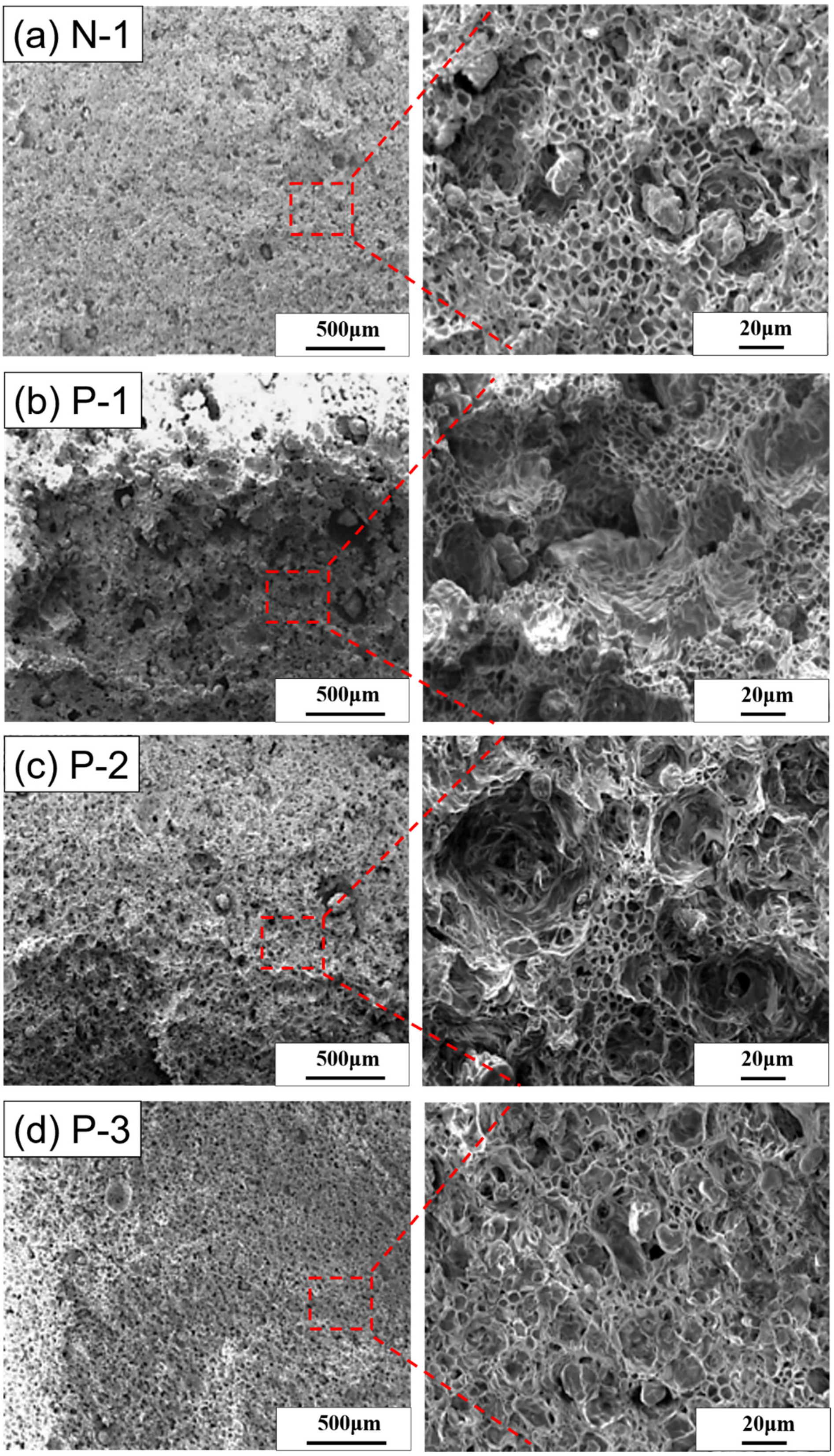

Figure S10. The SEM images of cell structure (a) [N-1], (b) [P-1], (c) [P-2], and (d)

[P-3] at the foaming temperature of 95 ℃ and the foaming pressure of 23.8 MPa.

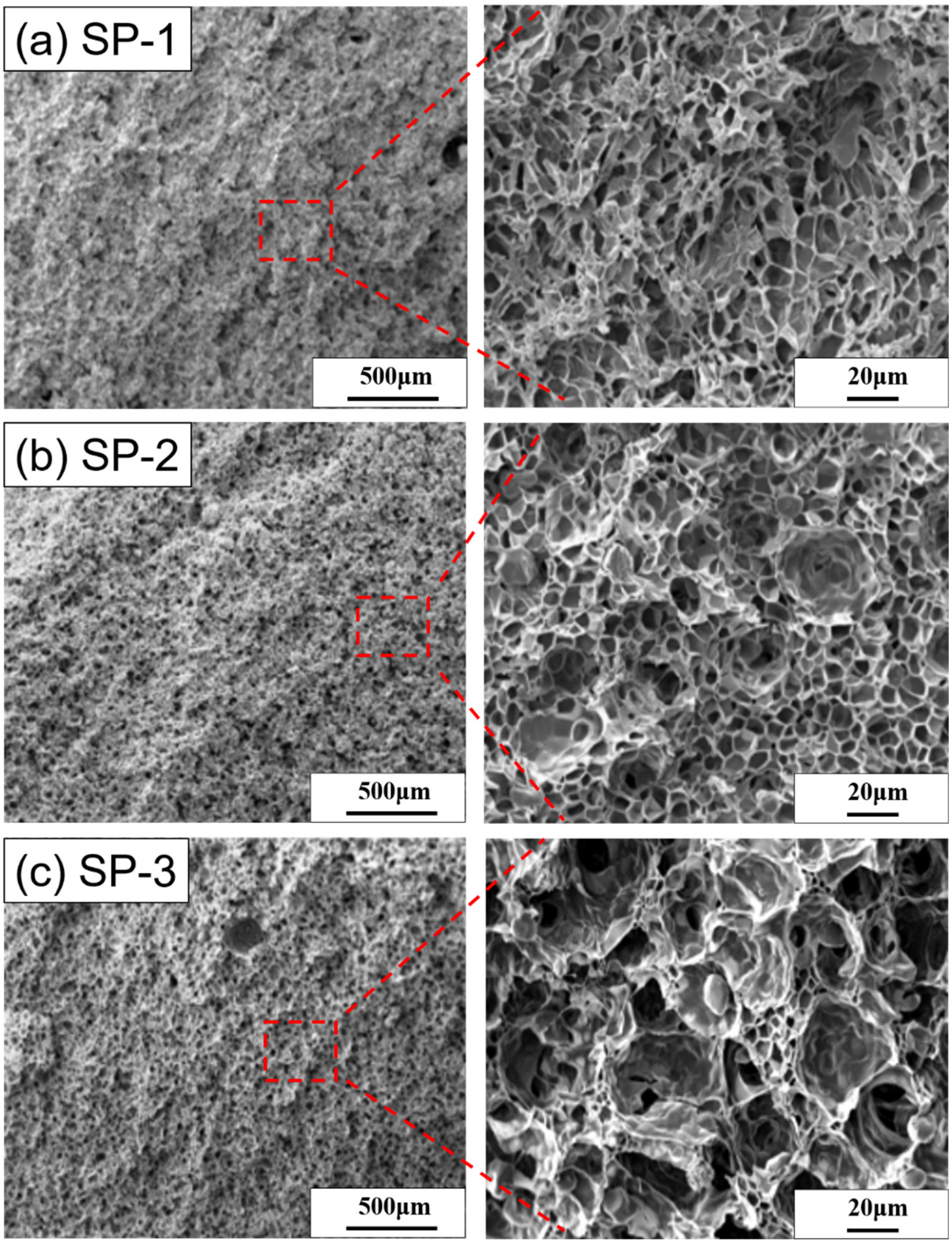

Figure S11. The SEM images of cell structure at the foaming temperature of 95 ℃ and the foaming pressure of 23.8 MPa. (a) [SP-1], (b) [SP-2], and (c) [SP-3].
